# Early Life Exposure to the Great Chinese Famine and Cardiometabolic Outcomes

**DOI:** 10.1001/jamanetworkopen.2025.45444

**Published:** 2025-11-25

**Authors:** Anne Cao, Zhixing Hong, Ning Liu, Jennifer Xiao, Douglas S. Lee, Calvin Ke

**Affiliations:** 1 Department of Medicine, Queen’s University, Kingston, Ontario, Canada; 2 ICES, Toronto, Ontario, Canada; 3 Institute of Health Policy, Management and Evaluation, Dalla Lana School of Public Health, University of Toronto, Toronto, Ontario, Canada; 4 Department of Medicine, Toronto General Hospital, University Health Network, Toronto, Ontario, Canada; 5 Department of Medicine, University of Toronto, Toronto, Ontario, Canada

## Abstract

**Importance:**

Epidemiologic studies suggest that people who were exposed to famine during early life but now live in nutrient-abundant environments have an elevated risk of type 2 diabetes (T2D) and other cardiometabolic conditions. Although many individuals who were exposed to famine in early life have now immigrated to high-income countries, the association between early life famine exposure and cardiometabolic outcomes among immigrants is unknown.

**Objective:**

To investigate the associations of early life exposure to the Great Chinese Famine with the incidence of T2D, hypertension, and cardiovascular hospitalization among immigrants.

**Design, Setting, and Participants:**

This population-based cohort study in Ontario, Canada, used 3 cohorts (1 for each coprimary outcome of T2D, hypertension, and cardiovascular hospitalization) of adult Chinese immigrants aged 20 to 85 years living in Ontario from April 1, 1992, to March 31, 2019. Participants were followed up until 85 years of age, relocation from Ontario, or March 31, 2023 (whichever occurred first). Data were analyzed from April 22, 2024, to September 30, 2025.

**Exposure:**

Prenatal, childhood, and adolescent exposure to famine, classified by year of birth (1941-1952). Those born before 1941 or after 1962 served as the comparison group.

**Main Outcomes and Measures:**

Incident T2D, hypertension, and cardiovascular hospitalization were the coprimary outcomes.

**Results:**

The T2D cohort included 188 292 individuals (exposed: mean [SD] age, 52.6 [10.8] years; 53.3% female; unexposed: mean [SD] age, 37.4 [12.5] years; 54.9% female), the hypertension cohort included 180 510 individuals (exposed: mean [SD] age, 51.8 [10.6] years; 52.3% female; unexposed: mean [SD] age, 36.6 [11.5] years; 55.0% female), and the cardiovascular hospitalization cohort included 208 921 individuals (exposed: mean [SD] age, 50.5 [12.0] years; 51.5% female; comparison: mean [SD] age, 37.8 [13.1] years; 54.6% female). Event rates among the famine-exposed group were 13.6% for T2D, 29.8% for hypertension, and 1.6% for cardiovascular hospitalization. Early life famine exposure was associated with increased hazards of T2D (prenatal: hazard ratio [HR], 1.58 [95% CI, 1.49-1.68]; childhood: HR, 1.45 [95% CI, 1.38-1.54]; adolescent: HR, 1.37 [95% CI, 1.28-1.46]) and hypertension (prenatal: HR, 1.22 [95% CI, 1.17-1.27]; childhood: HR, 1.25 [95% CI, 1.21-1.30]; adolescent: HR, 1.25 [95% CI, 1.20-1.31]). Prenatal and childhood exposure to famine were not associated with risk of cardiovascular hospitalization (prenatal: HR, 0.92 [95% CI, 0.76-1.12]; childhood: HR, 0.98 [95% CI, 0.86-1.11]), but adolescent exposure to famine was associated with a 14% decrease in risk of cardiovascular hospitalization (HR, 0.86 [95% CI, 0.75-0.98]).

**Conclusions and Relevance:**

In this cohort study of Chinese immigrants, early life famine exposure was strongly associated with increased risks of incident T2D and hypertension, but not cardiovascular hospitalization. Early life undernutrition is a novel cardiometabolic risk factor among immigrants associated with the burden of T2D and hypertension in high-income countries.

## Key Points

**Question:**

Is early life exposure to famine associated with increased risk of type 2 diabetes (T2D), hypertension, or cardiovascular hospitalization among Chinese immigrants?

**Findings:**

This cohort study of 208 921 adults found that prenatal, childhood, and adolescent exposure to famine were associated with 37% to 58% increased hazards of T2D and 22% to 25% increased hazards of hypertension. Prenatal and childhood famine exposures were not associated with cardiovascular hospitalization, while adolescent famine exposure was associated with a 14% decrease in cardiovascular hospitalization.

**Meaning:**

This study suggests that early life undernutrition is a novel cardiometabolic risk factor among immigrants that is associated with the burden of T2D and hypertension in high-income countries.

## Teaser

This cohort study comprising more than 200 000 Chinese immigrants investigates the associations of early life exposure to famine with the incidence of type 2 diabetes, hypertension, and cardiovascular hospitalization.

## Introduction

Epidemiologic studies suggest that people who were exposed to famine during early life but now live in nutrient-abundant environments have an elevated risk of type 2 diabetes (T2D) and other cardiometabolic conditions.^1,2,3,4^ The developmental origins of health and disease hypothesis suggests that the mismatch between the prenatal and later life environments is associated with cardiometabolic risk through organ developmental or epigenetic pathways.^5,6^ Similarly, the biological embedding concept suggests that childhood and adolescent undernutrition may be associated with these outcomes through pathways related to stress, insulin resistance, or gut microbial changes.^7^

The Great Chinese Famine (1959-1961) was the largest famine in human history, resulting in some 30 million deaths.^8^ Although some studies conducted in the People’s Republic of China (hereafter, *China*) suggested that early life exposure to this famine might increase later life cardiometabolic risk,^9,10,11^ most of these studies were cross-sectional,^9,12,13^ limited by small and nonrepresentative samples,^12,13,14,15^ and likely yielded spurious results due to age imbalances between the exposure and comparison groups.^12^ As ongoing undernutrition may avert the adverse cardiometabolic consequences of early life famine exposure,^16^ interpretation of these findings is also complicated by persistent undernutrition in some rural areas.^17^

Food insecurity is strongly associated with migration.^18^ Thus, many individuals who were exposed to famine in early life have now immigrated to high-income countries.^19^ These immigrants likely face a more extreme mismatch of their early and later life nutritional environments compared with their source populations. Therefore, the extent to which early life famine exposure is associated with cardiometabolic risk could be substantially greater than reported in prior epidemiologic studies, which were limited to nonmigrant populations. We aimed to investigate the associations of prenatal, childhood, and adolescent exposure to the Great Chinese Famine with the incidence of T2D, hypertension, and cardiovascular hospitalization among Canadian immigrants born in China. We hypothesized that early life famine exposure is associated with an increased risk of these cardiometabolic outcomes and that the magnitude of the association is greater than that observed in China.

## Methods

### Study Design and Setting

We conducted this population-based cohort study in Ontario, Canada, using administrative health databases as part of the Cardiovascular-Metabolic Disease in Asian Populations Relocated Abroad (CV-DIASPORA) study. Approximately 1.7 million Chinese people live in Canada, with nearly half residing in Ontario, the most populous province in Canada.^20^ The Ontario Health Insurance Plan provides universal, single-payer coverage of physician- and hospital-based health care services.^21^ We reported findings according to the Strengthening the Reporting of Observational Studies in Epidemiology (STROBE) reporting guideline for cohort studies. The use of the data in this project is authorized under section 45 of Ontario’s Personal Health Information Protection Act and does not require review by a research ethics board.

### Data Sources

We used the Immigration, Refugee and Citizenship Canada Permanent Resident Database, which contains records of immigrants arriving after 1985, to identify immigrants.^19^ We used the Ontario Diabetes Database to identify individuals with incident nongestational diabetes based on a highly specific validated algorithm (99.1% specificity).^22^ We applied another validated algorithm to exclude individuals with type 1 diabetes, and we classified the remainder as having T2D (98.5% positive predictive value [PPV] for T2D) (eTable 1 in Supplement 1).^23^ We used the Ontario Hypertension Dataset to identify incident hypertension, defined as 2 outpatient physician billing claims over a 3-year period (validated PPV, 87%) (eTable 1 in Supplement 1).^24^ We used the Canadian Institute for Health Information Discharge Abstract Database to identify cardiovascular hospitalizations. We used the Canadian census to identify household income in each individual’s neighborhood of residence (each comprising approximately 400-700 residents). These datasets were linked using unique encoded identifiers and analyzed at ICES.

### Study Population

We included adult residents of Ontario, aged 20 to 85 years, born in mainland China, with at least 2 years of Ontario Health Insurance Plan coverage, to provide a 2-year lookback window. We did not require a sampling strategy because our databases include all residents of Ontario. We created a separate cohort for each of the 3 outcomes (T2D, hypertension, and cardiovascular hospitalization) because different accrual periods were required to align with the various dates of data availability across these outcomes, and because exclusion criteria differed across cohorts. For the T2D and hypertension cohorts, we accrued individuals from April 1, 1994, to March 31, 2019, and respectively excluded people with prior T2D and hypertension. For the cardiovascular cohort, we accrued individuals from April 1, 1992, to March 31, 2019, and excluded people with prior cardiovascular hospitalization. The index date was defined as the earliest date during the accrual period in which the inclusion and exclusion criteria were met. We also excluded residents of long-term care facilities from all cohorts. Individuals could appear in multiple cohorts, as long as the cohort-specific inclusion and exclusion criteria were met.

### Exposure

Because the Great Chinese Famine occurred on a national scale and affected all provinces of mainland China,^12^ early life exposure to this famine is typically classified based on year of birth.^9,10,11,12,13,14,15^ This method has a PPV of approximately 84.8% compared with self-reported famine exposure ascertained by a nationally representative survey.^25^ We used the 3 categories defined in a similar study (eFigures 1-4 in Supplement 1): prenatal exposure (birth year: 1959-1962), childhood exposure (birth year: 1949-1958), and adolescent exposure (birth year: 1941-1948) to famine.^9^ Therefore, there was a total of 9 exposure groups across the 3 cohorts (T2D, hypertension, and cardiovascular hospitalization).

### Comparison

In epidemiologic famine studies, it is recommended that the comparison and exposure groups should have similar mean ages to avoid residual confounding by age.^12^ Because there were 9 exposure groups with different mean ages (according to the birth year definitions), we defined 9 different comparison groups with similar mean ages to the exposure groups. We achieved a balance of ages by including individuals born before 1941 or after 1962 in the comparison groups, and by adjusting age restrictions within each comparison group, based on published recommendations.^12^ Further details on the selection of these individuals are provided in the eAppendix and eTable 2 in Supplement 1.

### Outcomes and Covariates

The coprimary outcomes were incident T2D, hypertension, and cardiovascular hospitalization. Cardiovascular hospitalization was defined as having a hospitalization discharge abstract with the most responsible diagnosis of acute myocardial infarction (*International Classification of Diseases, Ninth Revision* [*ICD-9*] code 410; *International Statistical Classification of Diseases and Related Health Problems, Tenth Revision* [*ICD-10*] codes I21 and I22) or ischemic stroke (*ICD-9* codes 434, 436, and 3623; *ICD-10* codes I63 [excluding I636], I64, and H341). We followed individuals until the age of 85 years, relocation from Ontario, or March 31, 2023 (whichever occurred first). We included prespecified covariates based on clinical significance: sex, educational level, calendar year of immigration, immigration stream, and neighborhood income quintile.

### Statistical Analysis

We described the baseline characteristics using mean (SD) values. We constructed cause-specific proportional hazards models to calculate adjusted hazard ratios (HRs) estimating the associations of early life famine exposure with incidence of T2D, hypertension, and cardiovascular hospitalization, while accounting for the competing risk of death.^26^ Because each exposure group required a unique comparison group with a similar age distribution, we constructed 9 separate models: 1 model per exposure group, with 3 exposure groups per cohort, for 3 separate cohorts. Each model included a famine exposure group and its corresponding comparison group. We adjusted for the prespecified covariates listed. We tested for an interaction between famine exposure and each covariate listed, with adjustment for the other prespecified covariates. Missing socioeconomic status data were minimal (≤0.3%) and handled by complete-case analysis. We considered 2-sided *P* < .05 to be statistically significant. Data were analyzed from April 22, 2024, to September 30, 2025; analyses were performed with SAS, version 9.4 (SAS Institute Inc).

## Results

The T2D cohort included 188 292 individuals (exposed: mean [SD] age, 52.6 [10.8] years; 53.3% female and 46.7% male; unexposed: mean [SD] age, 37.4 [12.5] years; 54.9% female and 45.1% male), the hypertension cohort included 180 510 individuals (exposed: mean [SD] age, 51.8 [10.6]; 52.3% female and 47.7% male; unexposed: mean [SD] age, 36.6 [11.5] years; 55.0% female and 45.0% male), and the cardiovascular hospitalization cohort included 208 921 individuals (exposed: mean [SD] age, 50.5 [12.0] years; 51.5% female and 48.5% male; comparison: mean [SD] age, 37.8 [13.1] years; 54.6% female and 45.4% male) (Table).^19^ After defining the 3 exposure groups and 3 comparison groups within each of these cohorts (eFigures 2-4 in Supplement 1), each exposure group had a comparable mean age with its corresponding comparison group (≤3.1 years difference) (eTables 3-5 in Supplement 1). Most immigrants completed more than 12 years of schooling (ie, some postsecondary education and completed university; range, 51.8%-74.5%) (Table).^19^ There was a disproportionately high number of participants in the lowest income quintile (range, 27.5%-33.7%). Individuals with early life famine exposure were most commonly sponsored to immigrate by their family members living in Canada (range, 48.8%-52.5%), while most individuals without early life famine exposure immigrated through the economic stream (eg, businesspeople and skilled workers; range, 58.5%-61.7%). Most immigrants (41.7%-67.7%) arrived during the period from 1998 to 2008.

**Table.** Baseline Characteristics of the Study Cohorts^a^

Characteristic	Type 2 diabetes, No. (%)			Hypertension, No. (%)			Cardiovascular hospitalization, No. (%)		
	Early life famine exposure (n = 44 595)	No early life famine exposure (n = 143 697)	Early life famine exposure (n = 41 070)		No early life famine exposure (n = 139 440)	Early life famine exposure (n = 56 200)		No early life famine exposure (n = 152 721)
Age, mean (SD), y	52.6 (10.8)	37.4 (12.5)	51.8 (10.6)		36.6 (11.5)	50.5 (12.0)		37.8 (13.1)
Sex								
Female	23 791 (53.3)	78 827 (54.9)	21 473 (52.3)		76 691 (55.0)	28 950 (51.5)		83 374 (54.6)
Male	20 804 (46.7)	64 870 (45.1)	19 597 (47.7)		62 749 (45.0)	27 250 (48.5)		69 347 (45.4)
Educational level								
≤12 y of Schooling	21 501 (48.2)	37 723 (26.3)	19 301 (47.0)		35 597 (25.5)	26 665 (47.4)		42 580 (27.9)
Some postsecondary	9529 (21.4)	33 898 (23.6)	8813 (21.5)		33 011 (23.7)	12 404 (22.1)		36 041 (23.6)
Completed university	13 565 (30.4)	72 076 (50.2)	12 956 (31.5)		70 832 (50.8)	17 131 (30.5)		74 100 (48.5)
Immigration stream^b^								
Economic	17 157 (38.5)	86 619 (60.3)	16 802 (40.9)		85 992 (61.7)	22 686 (40.4)		89 286 (58.5)
Sponsored by family	23 428 (52.5)	45 952 (32.0)	20 456 (49.8)		42 551 (30.5)	27 423 (48.8)		51 072 (33.4)
Refugee	3257 (7.3)	10 129 (7.0)	3086 (7.5)		9936 (7.1)	3965 (7.1)		10 520 (6.9)
Income quintile								
1 (Lowest)	12 250 (27.5)	47 954 (33.4)	11 551 (28.1)		47 053 (33.7)	16 732 (29.8)		51 314 (33.6)
2	12 158 (27.3)	40 097 (27.9)	11 201 (27.3)		38 968 (27.9)	14 869 (26.5)		42 339 (27.7)
3	8102 (18.2)	23 404 (16.3)	7432 (18.1)		22 587 (16.2)	9755 (17.4)		24 825 (16.3)
4	6950 (15.6)	18 497 (12.9)	6245 (15.2)		17 688 (12.7)	8264 (14.7)		19 477 (12.8)
5 (Highest)	5076 (11.4)	13 285 (9.2)	4585 (11.2)		12 686 (9.1)	6249 (11.1)		14 080 (9.2)
Missing	59 (0.1)	460 (0.3)	56 (0.1)		458 (0.3)	331 (0.6)		686 (0.4)
Years of immigration								
1985-1997	7646 (17.1)	13 707 (9.5)	7502 (18.3)		13 098 (9.4)	16 469 (29.3)		20 668 (13.5)
1998-2008	22 503 (50.5)	96 592 (67.2)	21 288 (51.8)		94 367 (67.7)	23 451 (41.7)		97 725 (64.0)
2009-2019	14 446 (32.4)	33 398 (23.2)	12 280 (29.9)		31 975 (22.9)	16 280 (29.0)		34 328 (22.5)

^a^ Study cohorts include individuals born in mainland China who immigrated to Canada between 1985 and 2019. There were 9 statistical models (1 model per exposure group, 3 exposure groups per cohort, 3 cohorts in total). Each model included a subset of these cohorts: 1 famine exposure group defined by year of birth (prenatal [1959-1962], childhood [1949-1958], adolescent [1941-1948]), and a corresponding group with individuals selected to have a similar mean age to the exposure group. See eTables 3-5 in Supplement 1 for baseline characteristics of each exposure and comparison group.

^b^ Immigration stream is defined as the pathway through which individuals applied to enter Canada.^19^ Eligibility for each stream depends on skills, qualifications, and other circumstances. Economic immigrants (eg, business people, skilled workers) are evaluated by a rigorous point-based system that considers English or French language proficiency, Canadian and foreign work experience, age, educational level, technical skills, arranged employment, and other characteristics that deem them able to contribute to the Canadian economy. Sponsored family immigrants are relatives sponsored by a Canadian citizen or permanent resident, including but not limited to partners, parents, grandparents, or dependent children. The refugee stream is for those who are seeking protection or asylum in Canada due to persecution or other compassionate grounds, and may be referred by private sponsors or organizations such as the United Nations, or alternatively they may present their case on arrival as claimants.

### Type 2 Diabetes

**Figure 1.**
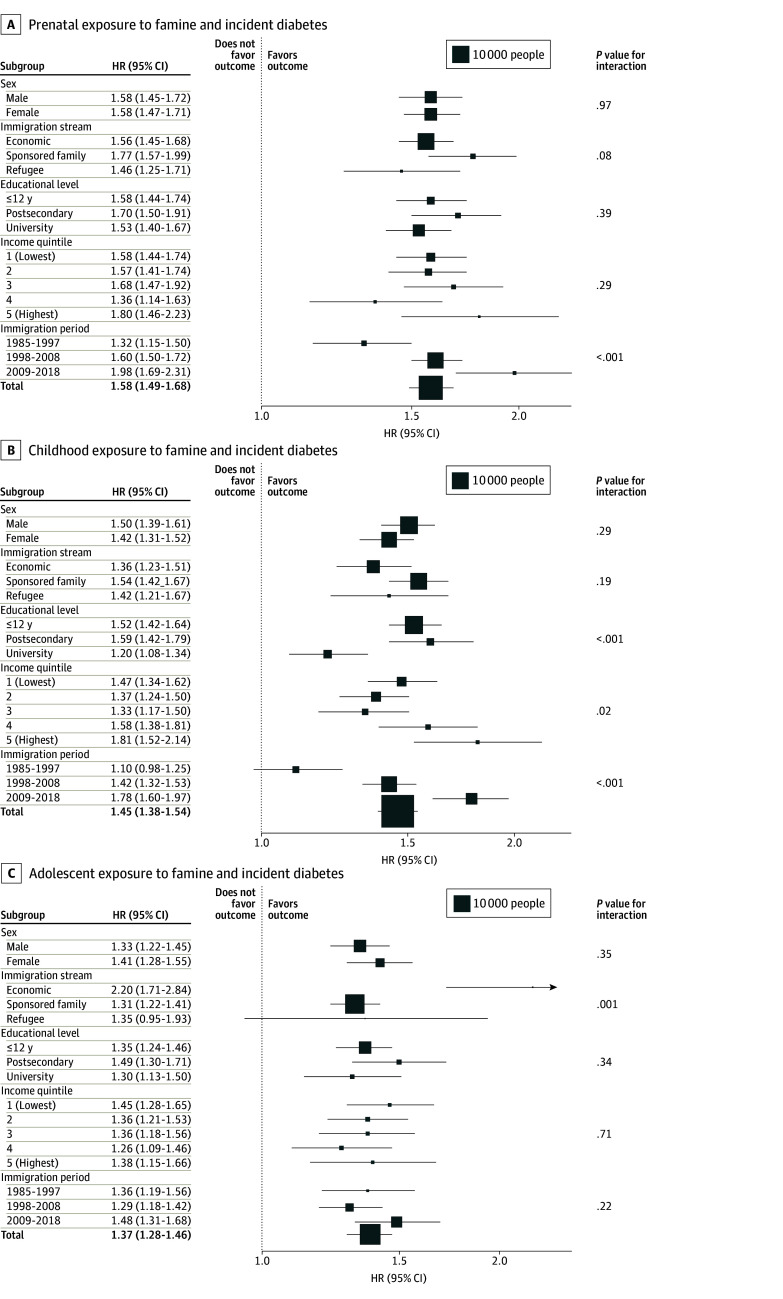
Associations of Prenatal, Childhood, and Adolescent Exposures to Famine With Incidence of Type 2 Diabetes The area of each box is proportional to the number of exposed individuals in each stratum. Estimates for each subgroup were generated by including an interaction term for famine exposure with the covariate; *P* values for interaction are shown in the rightmost column. HR indicates hazard ratio.

The event rate for T2D among the exposed group was 13.6% (6045 of 44 536). In the famine exposure cohort, the number of T2D events was 1523 of 13 627 (11.2%) in the prenatal group, 2694 of 18 971 (14.2%) in the childhood group, and 1828 of 11 938 (15.3%) in the adolescent group. In the comparison cohort, the number of T2D events was 6687 of 110 907 (6.0%) in the prenatal group, 3386 of 369 52 (9.2%) in the childhood group, and 2422 of 21 373 (11.3%) in the adolescent group. Prenatal famine exposure was associated with a 58% (adjusted HR, 1.58 [95% CI, 1.49-1.68]) higher risk of incident T2D, childhood famine exposure was associated with a 45% (adjusted HR, 1.45 [95% CI, 1.38-1.54]) higher risk of incident T2D, and adolescent famine exposure was associated with a 37% (adjusted HR, 1.37 [95% CI, 1.28-1.46]) higher risk of incident T2D (Figure 1; eFigure 5 and eTable 6 in Supplement 1). Adolescent famine exposure was more strongly associated with T2D among economic immigrants (eg, business people and skilled professionals; adjusted HR, 2.20 [95% CI, 1.71-2.84]) than those who were sponsored to immigrate by Canadian family members (sponsored family immigrants; adjusted HR, 1.31 [95% CI, 1.22-1.41]) and refugees (adjusted HR, 1.35 [95% CI, 0.95-1.93]; *P* = .001 for interaction). The association between childhood famine exposure and incident T2D varied by education (≤12 years: adjusted HR, 1.52 [95% CI, 1.42-1.64]; postsecondary: adjusted HR, 1.59 [95% CI, 1.42-.79]; university: adjusted HR, 1.20 [95% CI, 1.08-1.34]) (*P* < .001 for interaction) and income quintile (lowest: adjusted HR, 1.47 [95% CI, 1.34-1.62]; highest: adjusted HR, 1.81 [95% CI, 1.52-2.14]) (*P* = .02 for interaction), with the strongest point estimates observed among those with some postsecondary education and in the highest income quintile. The associations of prenatal and childhood famine exposure with incident T2D were stronger among those who immigrated more recently (prenatal: adjusted HR, 1.98 [95% CI, 1.69-2.31]; *P* < .001 for interaction; childhood: adjusted HR, 1.78 [95% CI, 1.60-1.97]; *P* < .001 for interaction).

### Hypertension

**Figure 2.**
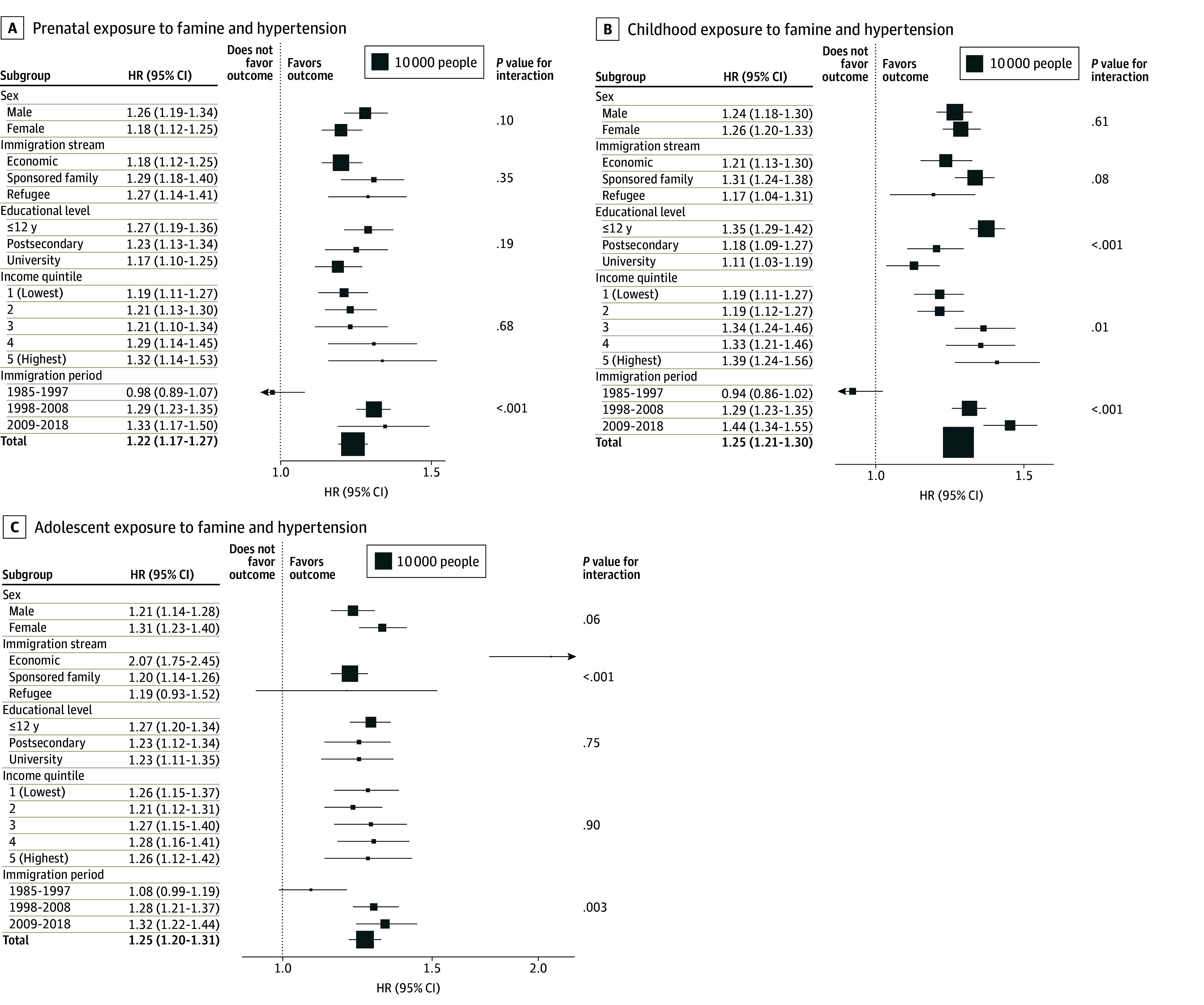
Associations of Prenatal, Childhood, and Adolescent Exposures to Famine With Incidence of Hypertension The area of each box is proportional to the number of exposed individuals in each stratum. Estimates for each subgroup were generated by including an interaction term for famine exposure with the covariate; *P* values for interaction are shown in the rightmost column. HR indicates hazard ratio.

The event rate for hypertension among the exposed group was 29.8% (12 236 of 41 014). In the famine exposure cohort, the number of hypertension events was 2938 of 13 363 (22.0%) in the prenatal group, 5443 of 17 696 (30.8%) in the childhood group, and 3855 of 9955 (38.7%) in adolescent group. In the comparison cohort, the number of hypertension events was 15 848 of 106 757 (14.8%) in the prenatal group, 7830 of 33 169 (23.6%) in the childhood group, and 5659 of 17 873 (31.7%) in the adolescent group. Prenatal, childhood, and adolescent famine exposure were associated with 22% to 25% increased hazards of incident hypertension (prenatal: adjusted HR, 1.22 [95% CI, 1.17-1.27]; childhood: adjusted HR, 1.25 [95% CI, 1.21-1.30]; adolescent: adjusted HR, 1.25 [95% CI, 1.20-1.31]) (Figure 2). Adolescent famine exposure was more strongly associated with incident hypertension among economic immigrants (adjusted HR, 2.07 [95% CI, 1.75-2.45]) than sponsored family immigrants (adjusted HR, 1.20 [95% CI, 1.14-1.26]) and refugees (adjusted HR, 1.19 [95% CI, 0.93-1.52]; *P* < .001 for interaction). Childhood famine exposure was more strongly associated with incident hypertension among those with 12 years or less of schooling (adjusted HR, 1.35 [95% CI, 1.29-1.42]) than those with higher levels of education (eg, university: adjusted HR, 1.11 [95% CI, 1.03-1.19]; *P* < .001 for interaction), and in higher vs lower income quintiles (highest quintile: adjusted HR, 1.39 [95% CI, 1.24-1.56]; *P* = .01 for interaction). Prenatal, childhood, and adolescent exposures to famine were most strongly associated with hypertension among individuals who immigrated more recently, from 2009 to 2018 (prenatal: adjusted HR, 1.33 [95% CI, 1.17-1.50]; *P* < .001 for interaction; childhood: adjusted HR, 1.44 [95% CI, 1.34-1.55]; *P* < .001 for interaction; adolescence: adjusted HR, 1.32 [95% CI, 1.22-1.44]; *P* = .003 for interaction).

### Cardiovascular Hospitalization

**Figure 3.**
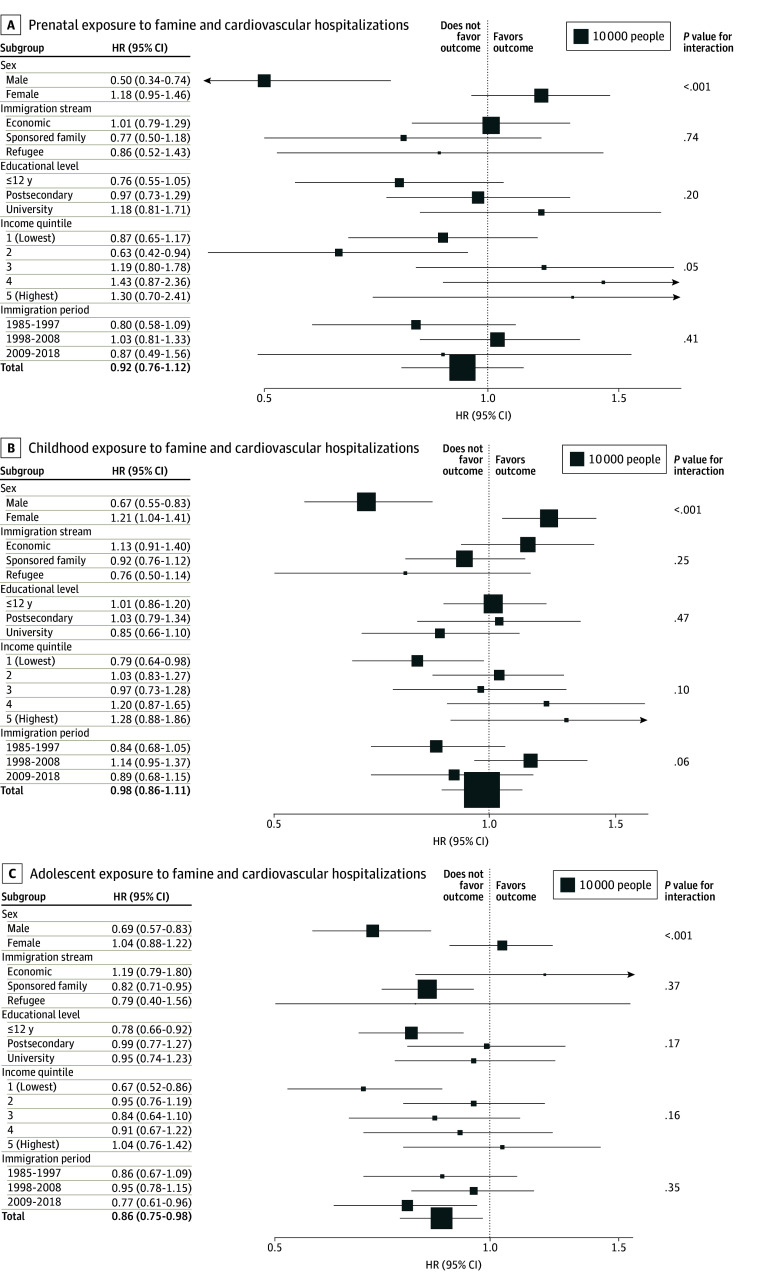
Associations of Prenatal, Childhood, and Adolescent Exposures to Famine With Incidence of Cardiovascular Hospitalization The area of each box is proportional to the number of exposed individuals in each stratum. Estimates for each subgroup were generated by including an interaction term for famine exposure with the covariate; *P* values for interaction are shown in the rightmost column. HR indicates hazard ratio.

The event rate for cardiovascular hospitalization among the exposed group was 1.6% (903 of 55 869). In the famine exposure cohort, the number of cardiovascular hospitalization events was 132 of 16 936 (0.8%) in the prenatal group, 380 of 24 500 (1.6%) in the childhood group, and 391 of 14 433 (2.7%) in adolescent group. In the comparison cohort, the number of cardiovascular hospitalization events was 1568 of 144 825 (1.1%) in the prenatal group, 1414 of 72 784 in the childhood group (1.9%), and 1194 of 25 162 (4.7%) in the adolescent group. Prenatal and childhood exposures to famine were not significantly associated with cardiovascular hospitalization (prenatal: adjusted HR, 0.92 [95% CI, 0.76-1.12] childhood: adjusted HR, 0.98 [95% CI, 0.86-1.11]), while adolescent exposure to famine was associated with a 14% decrease in the hazard of cardiovascular hospitalization (adjusted HR, 0.86 [95% CI, 0.75-0.98]) (Figure 3). There was a significant interaction of each famine exposure with sex, with relatively lower hazards of cardiovascular hospitalization among females (prenatal: adjusted HR, 0.50 [95% CI, 0.34-0.74]; childhood: adjusted HR, 0.67 [95% CI, 0.55-0.83]; adolescence: adjusted HR, 0.69 [95% CI, 0.57-0.83]; all *P* < .001 for interaction).

## Discussion

To our knowledge, this population-based cohort study is the first to specifically examine the outcomes of famine exposure among immigrants, and it is the largest to investigate incident cardiometabolic outcomes of early life exposure to the Great Chinese Famine. We showed that prenatal, childhood, and adolescent exposures to famine were strongly associated with increased risks of incident T2D and hypertension. The magnitude of the association between early life famine exposure and incident T2D (prenatal: adjusted HR, 1.58 [95% CI, 1.49-1.68]) is greater than that described in prior similar studies conducted in China.^27,28^ However, we found that early life exposure to famine was not associated with increased cardiovascular hospitalization among immigrants. We observed a 14% decreased hazard of cardiovascular hospitalization among the adolescent exposed group. This unexpected finding contrasts with prior studies^2,15,29^ and with our findings for T2D and hypertension. Although the reasons for this variation are unclear, immigrant selection, as well as differences in health systems and access to cardiovascular preventive care across countries, may play a role. These findings advance the literature supporting the concepts of the developmental origins of health and disease hypothesis and biological embedding by demonstrating that outcomes of famine exposure may manifest differently among immigrants compared with their source populations and that early life undernutrition is a novel cardiometabolic risk factor for immigrants that is associated with the burden of T2D and hypertension in high-income countries.^19^ Further work is required to address these gaps in cardiometabolic prevention.

We identified stronger associations between early life famine exposure and incident T2D compared with prior smaller studies of the Great Chinese Famine. A systematic review of 23 studies in China^12^ found that most were limited by a cross-sectional methodology and a lack of adjustment for age, with only 2 cohort studies examining incident T2D.^27,28^ In the China Kadoorie Biobank (n = 88 830), prenatal exposure to famine was associated with a 25% increase in the hazard of incident T2D (HR, 1.25 [95% CI, 1.07-1.45]).^27^ However, this study relied on routinely diagnosed T2D, which is likely undercaptured in China compared with Ontario.^30,31^ In the national China Cardiometabolic Disease and Cancer Cohort study (n = 77 925), prenatal exposure to famine was associated with a 17% increase (adjusted risk ratio, 1.17 [95% CI, 1.05-1.31]) in the risk of incident T2D systematically captured by oral glucose tolerance testing.^28^ However, the median follow-up time (3.6 years) and power were insufficient to detect any association with childhood famine exposure. By contrast, we demonstrated a relatively stronger association between prenatal vs later famine exposure with incident T2D—a pattern consistent with those with the highest level of self-reported hunger and weight loss during the Dutch famine from 1944 to 1945.^3^ Further research is required to understand the role of other factors, such as unhealthy diets, reduced physical activity, and sedentary environments, in generating the stronger associations among these immigrants relative to their source populations,^11,23^ as well as the variation across income quintiles, educational levels, immigration streams, and immigration time periods.

The observed association between early life famine exposure and incident hypertension was consistent with previous smaller cross-sectional studies. A prior systematic review identified 7 small and conflicting cross-sectional studies conducted in China.^13^ In the China Health and Nutrition Study (n = 1415), there was a positive association between childhood famine exposure and prevalent hypertension (adjusted odds ratio, 1.48 [95% CI, 1.11-1.98]), but power was insufficient to identify a significant association between prenatal famine exposure and prevalent hypertension (adjusted odds ratio, 1.24 [95% CI, 0.90-1.73]).^32^ Our study extends the literature by demonstrating robust and similar associations of prenatal, childhood, and adolescent famine exposures with incident hypertension—particularly in the highest income quintile for those exposed during childhood. These findings are consistent with a prior cross-sectional study demonstrating that exposure to famine during infancy was associated with prevalent hypertension in individuals with high income but not low income (adjusted odds ratio, 2.18 [95% CI, 1.14-4.18] vs 1.69 [95% CI, 0.90-3.19]) living in areas severely affected by the Great Chinese Famine.^33^ The observed associations are also consistent with an elevated risk of hypertension reported after exposure to other famines.^2,34^ However, the similarity in magnitude of the associations across prenatal, childhood, and adolescent famine exposures differed from the pattern observed with T2D, and further research is required to clarify the mechanisms explaining these patterns.

By contrast, the risk of incident cardiovascular hospitalization was not elevated among those with early life exposure to famine—a finding that differs from prior studies.^2,15,29^ In the China Kadoorie Biobank, prenatal famine exposure was associated with incident ischemic heart disease only among those with low physical activity (adjusted HR, 1.15 [95% CI, 1.05-1.26]) and with stroke only among those with low physical activity (adjusted HR, 1.13 [95% CI, 1.05-1.21]) or living in an urban area (adjusted HR, 1.18 [95% CI, 1.09-1.28]).^29^ However, childhood and adolescent famine exposures were not assessed. In the Kailuan community, only fetal (adjusted HR, 1.19 [95% CI, 1.04-1.38]), but not childhood (adjusted HR, 1.02 [95% CI, 0.84-1.23]), exposure to famine was associated with incident cardiovascular hospitalization.^15^ A much larger multicenter cross-sectional study of 259 657 adults across China found that the odds of self-reported cardiovascular disease appeared stronger among those exposed to famine during adolescence (adjusted odds ratio, 1.86 [95% CI, 1.42-2.44]) compared with childhood (adjusted odds ratio, 1.67 [95% CI, 1.37-2.03]) and the prenatal period (adjusted odds ratio, 1.35 [95% CI, 1.11-1.53]).^9^ Cardiovascular risk has also been associated with exposure to other famines.^2^ In particular, women with prenatal, childhood, or adolescent exposure to the Dutch famine had a higher risk of coronary heart disease (unadjusted HR, 1.38 [95% CI, 1.03-1.84]) but a lower risk of stroke (adjusted HR, 0.77 [95% CI, 0.59-0.99]).^4^ Further research is required to understand and confirm the observed 14% lower cardiovascular risk with adolescent famine exposure in our study and to determine whether routine identification of T2D and hypertension might have provided the opportunity for effective health care or behavior changes (eg, smoking cessation) to mitigate cardiovascular risk.^19,35^

### Strengths and Limitations

Our study has some strengths. To our knowledge, this study is the first to examine the risks of early life famine exposure specifically in immigrant populations and the largest cohort study examining incident cardiometabolic outcomes of the Great Chinese Famine. Our population-based methods ensured a large sample and minimal selection bias compared with prior survey-based studies, with virtually no loss to follow-up. The longitudinal nature of our study also allowed us to examine incident outcomes and avoided the recall bias inherent in previous cross-sectional studies.

However, this study also has some limitations. We lacked data on place of birth within mainland China and severity of famine exposure. We cannot exclude residual confounding due to unmeasured risk factors (eg, diet, physical activity, alcohol, and smoking). Prior evidence suggests more alcohol and tobacco use among famine-unexposed individuals, which would have attenuated the associations observed,^9^ although these individuals were younger than famine-exposed individuals. Further research is required to measure these risk factors in Chinese immigrants by famine exposure. Due to the observational nature of this study, we cannot rule out negative confounding due to exposure to other significant events in the comparison group (eg, war).

## Conclusions

In this cohort study, we found that early life exposure to the Great Chinese Famine was strongly associated with incident T2D and hypertension, but not cardiovascular hospitalization. Future studies might explore whether similar patterns are observed with other diasporic populations and famines. Furthermore, as Chinese immigrants continue to age, strategies to prevent T2D and hypertension may be especially useful and could be developed for those with a history of early life famine exposure. Similarly, proactive or more frequent screening to detect T2D and hypertension among exposed individuals may also be indicated. These findings emphasize the need for policymakers, health care professionals, and patients to consider the important early life influences that manifest decades later, contributing to the growing burden of T2D and hypertension worldwide.

## Notes

Supplement 1. **eTable 1.** Outcome Definitions **eFigure 1.** Classification of Famine Exposure and Comparison (Unexposed) Groups, According to Year of Birth **eAppendix.** Comparison Groups **eTable 2.** Range of Age at Index (Years) in the Exposure and Comparison Group **eFigure 2.** Flow Diagram of the Type 2 Diabetes Cohort **eFigure 3.** Flow Diagram of the Hypertension Cohort **eFigure 4.** Flow Diagram of the Cardiovascular Cohort **eTable 3.** Baseline Characteristics of the Type 2 Diabetes Cohort **eTable 4.** Baseline Characteristics of the Hypertension Cohort **eTable 5.** Baseline Characteristics of the Cardiovascular Cohort **eTable 6.** Crude (Unadjusted Hazard Ratios) of the Associations of Early Life Famine Exposure With Cardiometabolic Outcomes **eFigure 5.** Adjusted Cumulative Incidence Function Curves for Incident Type 2 Diabetes, Hypertension, and Cardiovascular Hospitalization
eReferences.

Supplement 2.
Data Sharing Statement
