# Supplementary material for: Early Life Exposure to the Great Chinese Famine and Cardiometabolic Outcomes
Source: JAMA Netw Open. 2025 Nov 25;8(11):e2545444. doi: 10.1001/jamanetworkopen.2025.45444 (PMC12648349; doi:10.1001/jamanetworkopen.2025.45444)
Supplement: Supplement 1. — eTable 1. Outcome Definitions eFigure 1. Classification of Famine Exposure and Comparison (Unexposed) Groups, According to Year of Birth eAppendix. Comparison Groups eTable 2. Range of Age at Index (Years) in the Exposure and Comparison Group eFigure 2. Flow Diagram of the Type 2 Diabetes Cohort eFigure 3. Flow Diagram of the Hypertension Cohort eFigure 4. Flow Diagram of the Cardiovascular Cohort eTable 3. Baseline Characteristics of the Type 2 Diabetes Cohort eTable 4. Baseline Characteristics of the Hypertension Cohort eTable 5. Baseline Characteristics of the Cardiovascular Cohort eTable 6. Crude (Unadjusted Hazard Ratios) of the Associations of Early Life Famine Exposure With Cardiometabolic Outcomes eFigure 5. Adjusted Cumulative Incidence Function Curves for Incident Type 2 Diabetes, Hypertension, and Cardiovascular Hospitalization eReferences. [file jamanetwopen-e2545444-s001.pdf]

Supplementary Online Content

Cao A, Hong Z, Liu N, Xiao J, Lee DS, Ke C. Early life exposure to the Great Chinese Famine and cardiometabolic outcomes. *JAMA Netw Open.* 2025;8(11):e2545444. doi:10.1001/jamanetworkopen.2025.45444

eTable 1. Outcome Definitions

eFigure 1. Classification of Famine Exposure and Comparison (Unexposed) Groups, According to Year of Birth

eAppendix. Comparison Groups

eTable 2. Range of Age at Index (Years) in the Exposure and Comparison Group

eFigure 2. Flow Diagram of the Type 2 Diabetes Cohort

eFigure 3. Flow Diagram of the Hypertension Cohort

eFigure 4. Flow Diagram of the Cardiovascular Cohort

eTable 3. Baseline Characteristics of the Type 2 Diabetes Cohort

eTable 4. Baseline Characteristics of the Hypertension Cohort

eTable 5. Baseline Characteristics of the Cardiovascular Cohort

eTable 6. Crude (Unadjusted Hazard Ratios) of the Associations of Early Life Famine Exposure With Cardiometabolic Outcomes

eFigure 5. Adjusted Cumulative Incidence Function Curves for Incident Type 2 Diabetes, Hypertension, and Cardiovascular Hospitalization

eReferences.

This supplementary material has been provided by the authors to give readers additional information about their work.

eTable 1. Outcome Definitions

Condition	Definition
Type 2 Diabetes	Type 2 diabetes is defined using a **2-step algorithm** (illustrated in Supplementary Figure 1):<br>1. Identify all individuals in Ontario with **non-gestational diabetes (any type)** using the Ontario Diabetes Database specific definition listed below, which has a validated specificity of 99.2% and positive predictive value of 92.5%:<br>• Definition: 3 physician claims to the Ontario Health Insurance Plan with International Classification of Diseases (ICD)-9 code 250 within 1 year. The Ontario Health Insurance Plan does not use ICD-10 codes.<br>2. Out of those identified in step 1, we **exclude all individuals with type 1 diabetes**, which is defined as fulfilling any of the criteria listed below. The **remaining individuals are defined as having type 2 diabetes**. This definition has a validated sensitivity of 99.3% and positive predictive value of 98.5% for type 2 diabetes.<br>• 4 physician claim codes for ICD-9 250 in any 1 year at age <18 years; or<br>• enrolment in the Ontario Ministry of Health Assistive Device Program for insulin pump; or<br>• inpatient admission or emergency department visit for diabetic ketoacidosis at age <18 years (ICD9 code: 2501; ICD-10: E100, E101, E110, E111, E130, E131, E140, E141)
Hypertension	Hypertension is defined by 2 outpatient physician billing claims over a 3-year period for any of the ICD codes listed below:<br>ICD-9: 401, 402, 403, 404, 405; ICD-10: I10, I11, I12, I13, I15
Acute myocardial infarction	ICD-9: 410; ICD-10: I21, I22
Ischemic stroke	ICD-9: 434 436, 3623; ICD-10: I63 I64

© 2025 Cao A et al. *JAMA Network Open.*

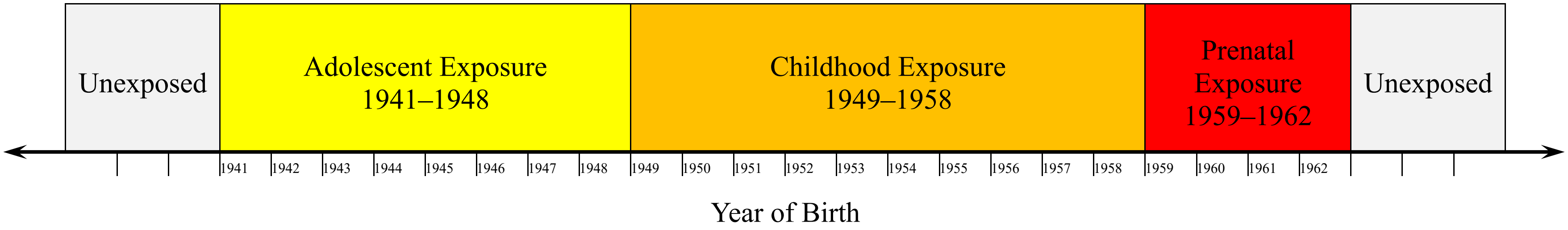

eFigure 1. Classification of Famine Exposure and Comparison (Unexposed) Groups, According to Year of Birth

Because the Great Chinese Famine occurred on a national scale and affected all provinces of mainland China,[1] early life exposure to this famine is typically classified based on year of birth.[1–7] This method has a PPV of around 84.8% compared to self-reported famine exposure ascertained by a nationally-representative survey.[8] We used the 3 standard categories defined in a similar study: prenatal exposure (birth year: 1959–1962), childhood exposure (birth year: 1949–1958), and adolescent exposure (birth year: 1941–1948) to famine.[2]

© 2025 Cao A et al. *JAMA Network Open.*

eAppendix. Comparison Groups

Based on the findings of a previous meta-analysis, it is recommended that the comparison group should include individuals born both before and after the famine, such that the mean age of the comparison group is similar to that of the exposure group, to minimize residual confounding by age.[1] Therefore, individuals born before 1941 and after 1962 were eligible for inclusion in the comparison group. However, the mean age of this pool of individuals was much lower than the exposure groups, due to an excess of younger individuals born after 1962. Therefore, we restricted each comparison group by setting a minimum age at index to yield a mean age similar to the corresponding exposure group. For example, the mean age of the prenatal exposure group in the type 2 diabetes (T2D) cohort was 42.9 years. Thus, we restricted the comparison group by excluding those aged 25–29 years at index, resulting in the inclusion of those aged 30–85 years at index, and yielding a mean age of 40.6 years. As the mean age of each exposure group differed according to their years of birth, we repeated this procedure to create a total of 9 comparison groups corresponding to the 9 exposure groups (3 exposure groups per cohort, 3 cohorts in total), setting the minimum age at index to the nearest multiple of 5. Individuals could appear in multiple comparison groups, as long as cohort-specific inclusion and exclusion criteria were met. The ranges of age at index date for all 9 comparison groups are shown below:

© 2025 Cao A et al. *JAMA Network Open.*

eTable 2. Range of Age at Index (Years) in the Exposure and Comparison Groups

	Cohort		
	Type 2 Diabetes	Hypertension	Cardiovascular
Prenatal exposed group	30–64	30–64	25–64
Prenatal comparison group	30–85	30–85	25–85
Childhood exposed group	35–74	35–74	30–74
Childhood comparison group	40–85	40–85	35–85
Adolescent exposed group	45–79	45–79	40–79
Adolescent comparison group	45–85	45–85	45–85

© 2025 Cao A et al. *JAMA Network Open.*

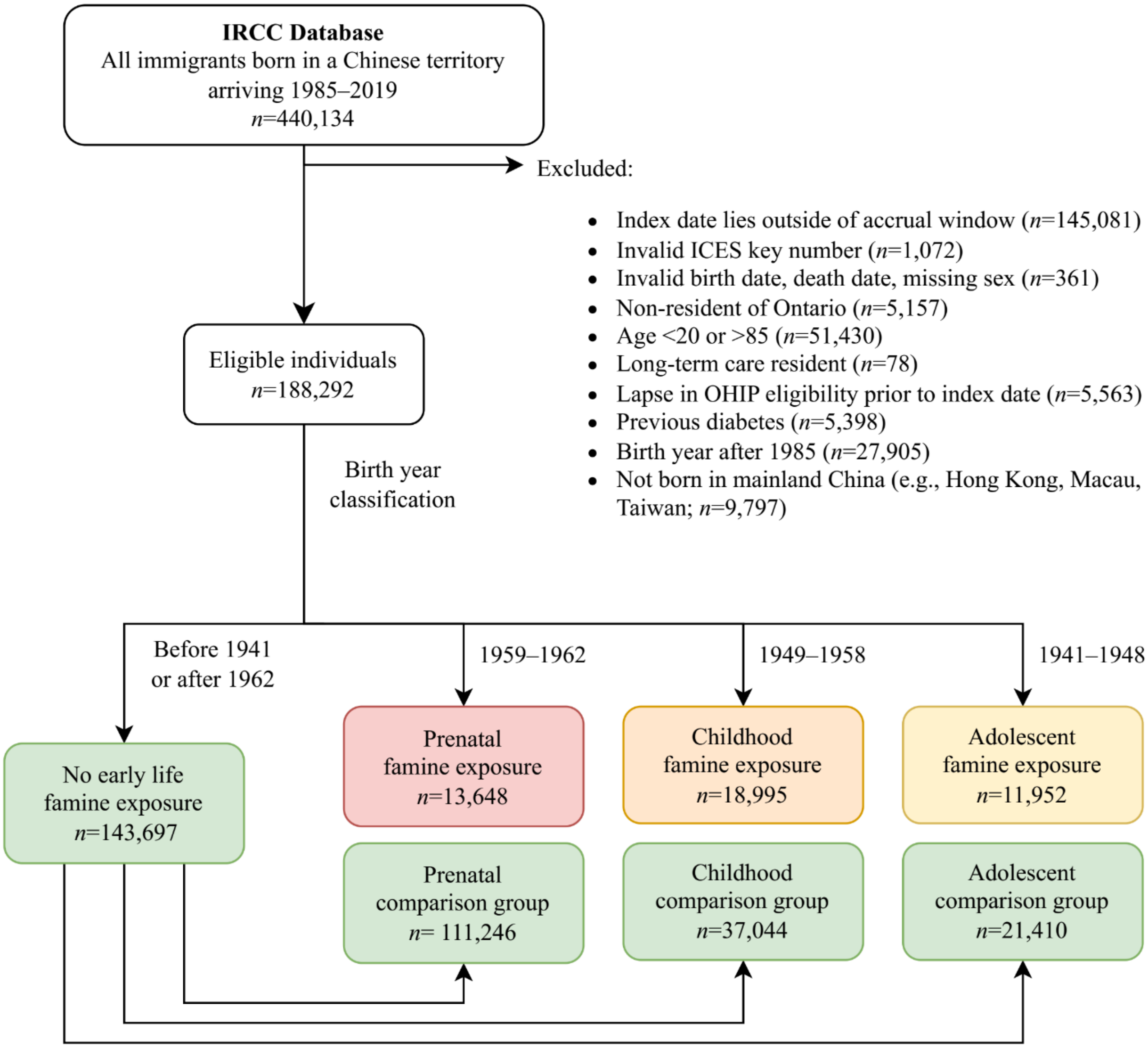

eFigure 2. Flow Diagram of the Type 2 Diabetes Cohort

We created a separate cohort for each study outcome because different accrual periods were required to align with the various dates of data availability across these outcomes. For the type 2 diabetes outcome, the earliest availability of data was April 1, 1994. Therefore, the accrual window was defined as April 1, 1994 to March 31, 2019. We classified early life exposure to the Great Chinese Famine based on year of birth (see eFigure 4): prenatal exposure (birth year: 1959–1962), childhood exposure (birth year: 1949–1958), and adolescent exposure (birth year: 1941–1948) to famine. Therefore, there were 3 exposure groups within this cohort. In epidemiological famine studies, it is recommended that each exposure group should have a comparison groups with a similar mean age, to avoid residual confounding by age. Because the 3

© 2025 Cao A et al. *JAMA Network Open.*

exposure groups had different mean ages, we therefore defined 3 different comparison groups. Individuals born before 1941 or after 1962 were eligible to be included in each comparison group. A subset of individuals within this pool were selected for each comparison group. Individuals could appear in multiple comparison groups (for further details on this procedure, please see Supplement page 4). The ICES key number is a unique encoded identifier for data linkage.

Abbreviations: IRCC, Immigration, Refugee and Citizenship Canada; OHIP, Ontario Health Insurance Plan

© 2025 Cao A et al. *JAMA Network Open.*

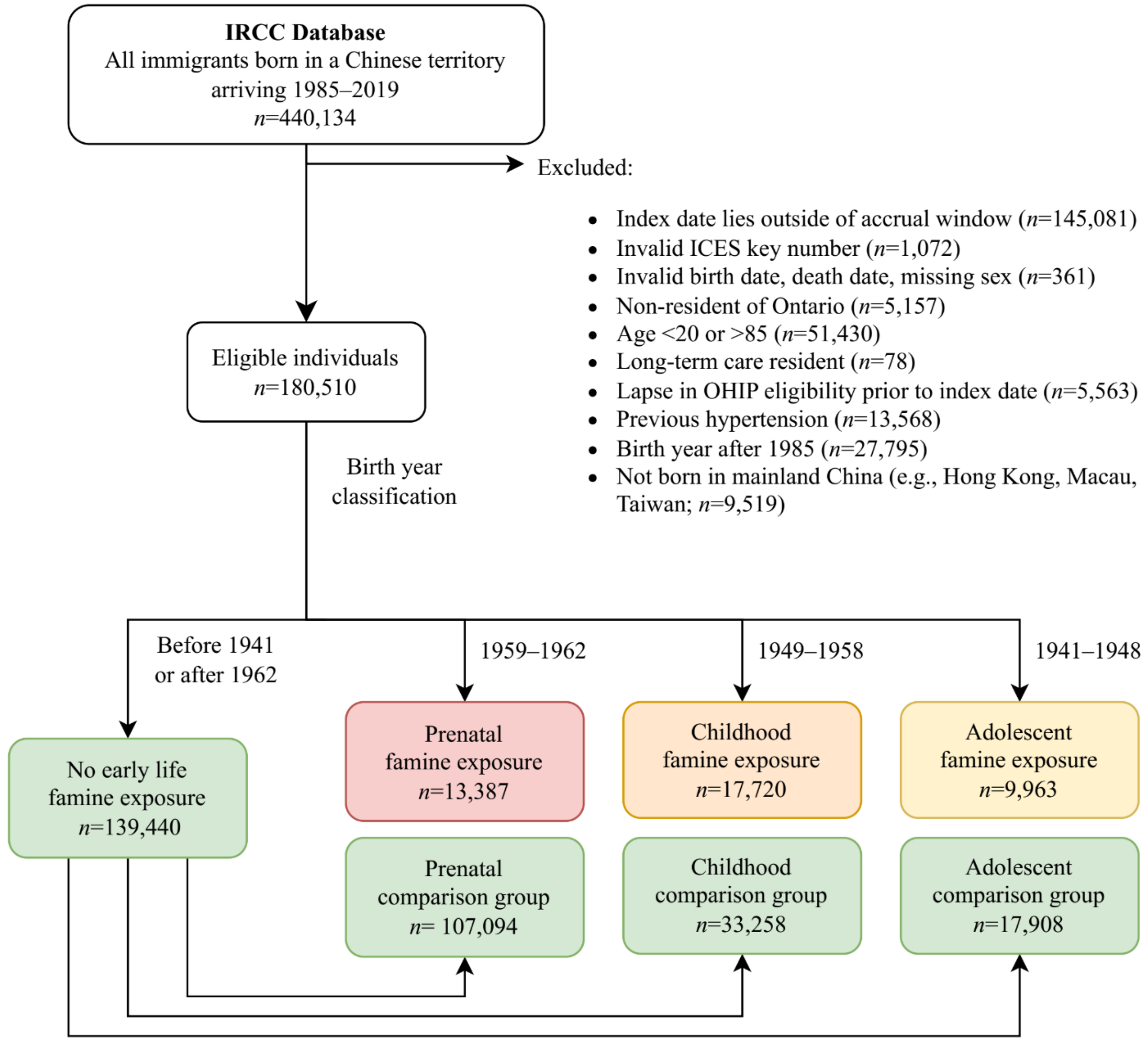

eFigure 3. Flow Diagram of the Hypertension Cohort

We created a separate cohort for each study outcome because the earliest availability of data differed across the outcomes. For the hypertension outcome, the earliest availability of data was April 1, 1994. Therefore, the accrual window was defined as April 1, 1994 to March 31, 2019.

Abbreviations: IRCC, Immigration, Refugee and Citizenship Canada; OHIP, Ontario Health Insurance Plan

© 2025 Cao A et al. *JAMA Network Open.*

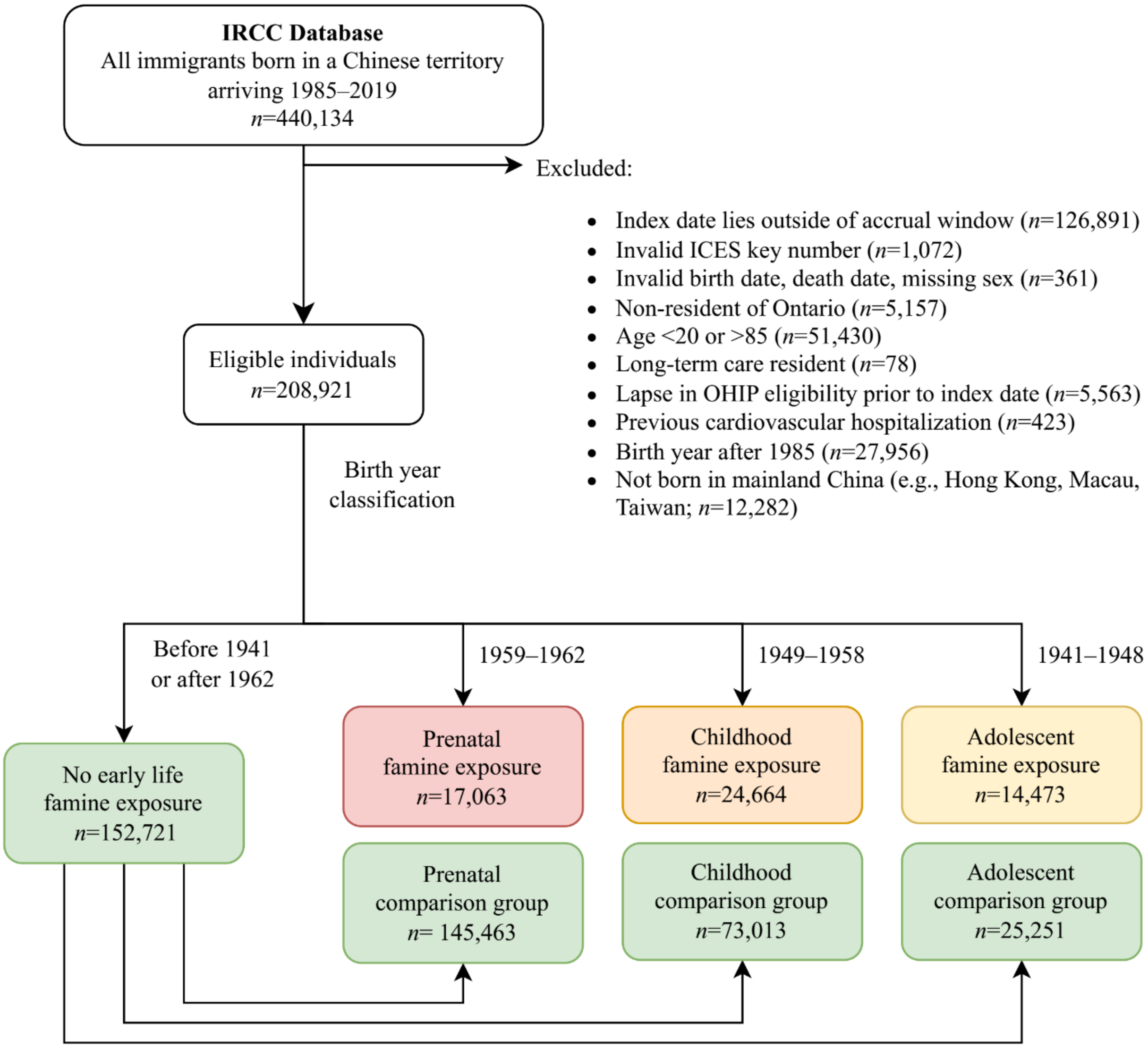

eFigure 4. Flow Diagram of the Cardiovascular Cohort

We created a separate cohort for each study outcome because the earliest availability of data differed across the outcomes. For the cardiovascular hospitalization outcome, the earliest availability of data was April 1, 1992. Therefore, the accrual window was defined as April 1, 1992 to March 31, 2019.

Abbreviations: IRCC, Immigration, Refugee and Citizenship Canada; OHIP, Ontario Health Insurance Plan

© 2025 Cao A et al. *JAMA Network Open.*

eTable 3. Baseline Characteristics of the Type 2 Diabetes Cohort

The type 2 diabetes cohort included individuals born in mainland China who immigrated to Canada between 1985 to 2019. Early life famine exposure was classified according to year of birth: prenatal (1959–1962), childhood (1949–1958), adolescent (1941–1948), and unexposed (before 1949 or after 1962). Each exposure group had a comparison group with a similar mean age (see Supplement page 4 for details). All values are counts and percentages unless otherwise stated.

	Prenatal famine exposure	Prenatal comparison group	Childhood famine exposure	Childhood comparison group	Adolescent famine exposure	Adolescent comparison group
	N=13,648	N=111,246	N=18,995	N=37,044	N=11,952	N=21,410
Age (years; mean, standard deviation)	42.9 (5.7)	40.6 (12.4)	52.2 (8.2)	53.8 (13.8)	64.5 (6.7)	62.6 (11.9)
Female	6,664 (48.8)	58,757 (52.8)	10,138 (53.4)	19,096 (51.5)	6,989 (58.5)	10,914 (51.0)
Education						
≤12 years of schooling	4,150 (30.4)	26,582 (23.9)	10,326 (54.4)	14,425 (38.9)	7,025 (58.8)	10,418 (48.7)
Some post-secondary	2,899 (21.2)	24,146 (21.7)	4,010 (21.1)	8,433 (22.8)	2,620 (21.9)	4,896 (22.9)
Completed university	6,599 (48.4)	60,518 (54.4)	4,659 (24.5)	14,186 (38.3)	2,307 (19.3)	6,096 (28.5)
Immigration stream*						
Economic	9,368 (68.6)	72,184 (64.9)	7,320 (38.5)	15,706 (42.4)	469 (3.9)	4,428 (20.7)
Sponsored Family	2,742 (20.1)	30,844 (27.7)	9,569 (50.4)	17,949 (48.5)	11,117 (93.0)	15,588 (72.8)
Refugee	1,323 (9.7)	7,520 (6.8)	1,656 (8.7)	3,086 (8.3)	278 (2.3)	1,204 (5.6)
Income Quintile						
1 (lowest)	4,799 (35.2)	37,171 (33.4)	5,306 (27.9)	9,328 (25.2)	2,145 (17.9)	4,702 (22.0)
2	3,837 (28.1)	29,730 (26.7)	5,516 (29.0)	9,620 (26.0)	2,805 (23.5)	5,424 (25.3)
3	2,333 (17.1)	17,955 (16.1)	3,402 (17.9)	6,468 (17.5)	2,367 (19.8)	3,868 (18.1)
4	1,547 (11.3)	14,869 (13.4)	2,796 (14.7)	6,185 (16.7)	2,607 (21.8)	3,937 (18.4)
5 (highest)	1,111 (8.1)	11,182 (10.1)	1,951 (10.3)	5,351 (14.4)	2,014 (16.9)	3,442 (16.1)
Missing	21 (0.2)	339 (0.3)	24 (0.1)	92 (0.2)	14 (0.1)	37 (0.2)
Year of Immigration						

© 2025 Cao A et al. *JAMA Network Open.*

1985–1997	2,812 (20.6)	8,863 (8.0)	3,608 (19.0)	3,916 (10.6)	1,226 (10.3)	3,871 (18.1)
1998–2008	9,021 (66.1)	73,416 (66.0)	8,954 (47.1)	18,508 (50.0)	4,528 (37.9)	8,525 (39.8)
2009–2019	1,815 (13.3)	28,967 (26.0)	6,433 (33.9)	14,620 (39.5)	6,198 (51.9)	9,014 (42.1)
Hypertension	508 (3.7)	5,633 (5.1)	2,036 (10.7)	4,970 (13.4)	2,892 (24.2)	4,503 (21.0)

© 2025 Cao A et al. *JAMA Network Open.*

eTable 4. Baseline Characteristics of the Hypertension Cohort

The hypertension cohort included individuals born in mainland China who immigrated to Canada between 1985 to 2019. Early life famine exposure was classified according to year of birth: prenatal (1959–1962), childhood (1949–1958), adolescent (1941–1948), and unexposed (before 1949 or after 1962). Each exposure group had a comparison group with a similar mean age (see Supplement page 4 for details). All values are counts and percentages unless otherwise stated.

	Prenatal famine exposure	Prenatal comparison group	Childhood famine exposure	Childhood comparison group	Adolescent famine exposure	Adolescent comparison group
	N=13,387	N=107,094	N=17,720	N=33,258	N=9,963	N=17,908
Age (years; mean, standard deviation)	42.8 (5.6)	39.7 (11.4)	51.8 (8.1)	52.2 (13.1)	64.1 (6.9)	61.1 (12.0)
Female	6,514 (48.7)	56,669 (52.9)	9,287 (52.4)	17,116 (51.5)	5,672 (56.9)	9,065 (50.6)
Education						
≤12 years of schooling	4,010 (30.0)	24,489 (22.9)	9,434 (53.2)	12,441 (37.4)	5,857 (58.8)	8,537 (47.7)
Some post-secondary	2,848 (21.3)	23,291 (21.7)	3,774 (21.3)	7,644 (23.0)	2,191 (22.0)	4,173 (23.3)
Completed university	6,529 (48.8)	59,314 (55.4)	4,512 (25.5)	13,173 (39.6)	1,915 (19.2)	5,198 (29.0)
Immigration stream*						
Economic	9,233 (69.0)	71,585 (66.8)	7,123 (40.2)	15,368 (46.2)	446 (4.5)	4,292 (24.0)
Sponsored Family	2,644 (19.8)	27,515 (25.7)	8,615 (48.6)	14,676 (44.1)	9,197 (92.3)	12,352 (69.0)
Refugee	1,297 (9.7)	7,332 (6.8)	1,549 (8.7)	2,942 (8.8)	240 (2.4)	1,101 (6.1)
Income Quintile						
1 (lowest)	4,732 (35.3)	36,291 (33.9)	4,997 (28.2)	8,574 (25.8)	1,822 (18.3)	4,015 (22.4)
2	3,741 (27.9)	28,646 (26.7)	5,134 (29.0)	8,660 (26.0)	2,326 (23.3)	4,541 (25.4)
3	2,293 (17.1)	17,162 (16.0)	3,186 (18.0)	5,723 (17.2)	1,953 (19.6)	3,184 (17.8)
4	1,519 (11.3)	14,074 (13.1)	2,579 (14.6)	5,433 (16.3)	2,147 (21.5)	3,234 (18.1)
5 (highest)	1,078 (8.1)	10,584 (9.9)	1,800 (10.2)	4,779 (14.4)	1,707 (17.1)	2,899 (16.2)
Missing income	24 (0.2)	337 (0.3)	24 (0.1)	89 (0.3)	8 (0.1)	35 (0.2)
Year of Immigration						

© 2025 Cao A et al. *JAMA Network Open.*

1985–1997	2,799 (20.9)	8,259 (7.7)	3,552 (20.0)	3,325 (10.0)	1,151 (11.6)	3,285 (18.3)
1998–2008	8,902 (66.5)	71,274 (66.6)	8,502 (48.0)	16,641 (50.0)	3,884 (39.0)	6,841 (38.2)
2009–2019	1,686 (12.6)	27,561 (25.7)	5,666 (32.0)	13,292 (40.0)	4,928 (49.5)	7,782 (43.5)
Type 2 diabetes	177 (1.3)	1,102 (1.0)	585 (3.3)	912 (2.7)	726 (7.3)	780 (4.4)

© 2025 Cao A et al. *JAMA Network Open.*

eTable 5. Baseline Characteristics of the Cardiovascular Cohort

The cardiovascular cohort included individuals born in mainland China who immigrated to Canada between 1985 to 2019. Early life famine exposure was classified according to year of birth: prenatal (1959–1962), childhood (1949–1958), adolescent (1941–1948), and unexposed (before 1949 or after 1962). Each exposure group had a comparison group with a similar mean age (see Supplement page 4 for details). All values are counts and percentages unless otherwise stated.

	Prenatal famine exposure	Prenatal comparison group	Childhood famine exposure	Childhood comparison group	Adolescent famine exposure	Adolescent comparison group
	N=17,063	N=145,463	N=24,664	N=73,013	N=14,473	N=25,251
Age (years; mean, standard deviation)	40.8 (7.00)	38.6 (13.0)	49.6 (9.6)	47.0 (13.7)	63.3 (8.1)	63.1 (11.6)
Female	8,160 (47.8)	79,155 (54.4)	12,594 (51.1)	37,584 (51.5)	8,196 (56.6)	12,926 (51.2)
Education						
≤12 years of schooling	5,570 (32.6)	37,379 (25.7)	12,883 (52.2)	22,101 (30.3)	8,212 (56.7)	12,803 (50.7)
Some post-secondary	3,724 (21.8)	34,260 (23.6)	5,425 (22.0)	15,781 (21.6)	3,255 (22.5)	5,705 (22.6)
Completed university	7,769 (45.5)	73,824 (50.8)	6,356 (25.8)	35,131 (48.1)	3,006 (20.8)	6,743 (26.7)
Immigration stream*						
Economic	11,124 (65.2)	87,753 (60.3)	10,245 (41.5)	41,449 (56.8)	1,317 (9.1)	4,888 (19.4)
Sponsored Family	3,626 (21.3)	46,828 (32.2)	11,181 (45.3)	25,403 (34.8)	12,616 (87.2)	18,835 (74.6)
Refugee	1,581 (9.3)	9,432 (6.5)	2,036 (8.3)	5,651 (7.7)	348 (2.4)	1,290 (5.1)
Income Quintile						
1 (lowest)	6,312 (37.0)	48,850 (33.6)	7,593 (30.8)	22,210 (30.4)	2,827 (19.5)	5,882 (23.3)
2	4,632 (27.1)	39,921 (27.4)	6,877 (27.9)	19,078 (26.1)	3,360 (23.2)	6,404 (25.4)
3	2,773 (16.3)	23,573 (16.2)	4,186 (17.0)	12,271 (16.8)	2,796 (19.3)	4,527 (17.9)
4	1,839 (10.8)	18,779 (12.9)	3,392 (13.8)	10,662 (14.6)	3,033 (21.0)	4,479 (17.7)
5 (highest)	1,380 (8.1)	13,702 (9.4)	2,452 (9.9)	8,563 (11.7)	2,417 (16.7)	3,870 (15.3)
Missing income	127 (0.7)	638 (0.4)	164 (0.7)	229 (0.3)	40 (0.3)	89 (0.4)
Year of Immigration						

© 2025 Cao A et al. *JAMA Network Open.*

1985–1997	5,893 (34.5)	17,836 (12.3)	8,190 (33.2)	7,306 (10.0)	2,386 (16.5)	6,317 (25.0)
1998–2008	9,244 (54.2)	93,359 (64.2)	9,343 (37.9)	43,933 (60.2)	4,864 (33.6)	9,222 (36.5)
2009–2019	1,926 (11.3)	34,268 (23.6)	7,131 (28.9)	21,774 (29.8)	7,223 (49.9)	9,712 (38.5)
Type 2 diabetes	217 (1.3)	1,809 (1.2)	823 (3.3)	1,712 (2.3)	1,166 (8.1)	1,424 (5.6)
Hypertension	557 (3.3)	6,776 (4.7)	2,329 (9.4)	6,352 (8.7)	3,408 (23.5)	5,450 (21.6)

© 2025 Cao A et al. *JAMA Network Open.*

eTable 6. Crude (Unadjusted Hazard Ratios) of the Associations of Early Life Famine Exposure With Cardiometabolic Outcomes

	Prenatal	Childhood	Adolescent
Type 2 Diabetes	1.55 (1.47–1.64)	1.32 (1.26–1.40)	1.48 (1.39–1.57)
Hypertension	1.28 (1.23–1.33)	1.10 (1.06–1.14)	1.28 (1.23–1.34)
Cardiovascular hospitalization	0.48 (0.40–0.57)	0.52 (0.47–0.59)	0.60 (0.54–0.68)

Results were generated using cause-specific proportional hazards models, with death considered as a competing event.

© 2025 Cao A et al. *JAMA Network Open.*

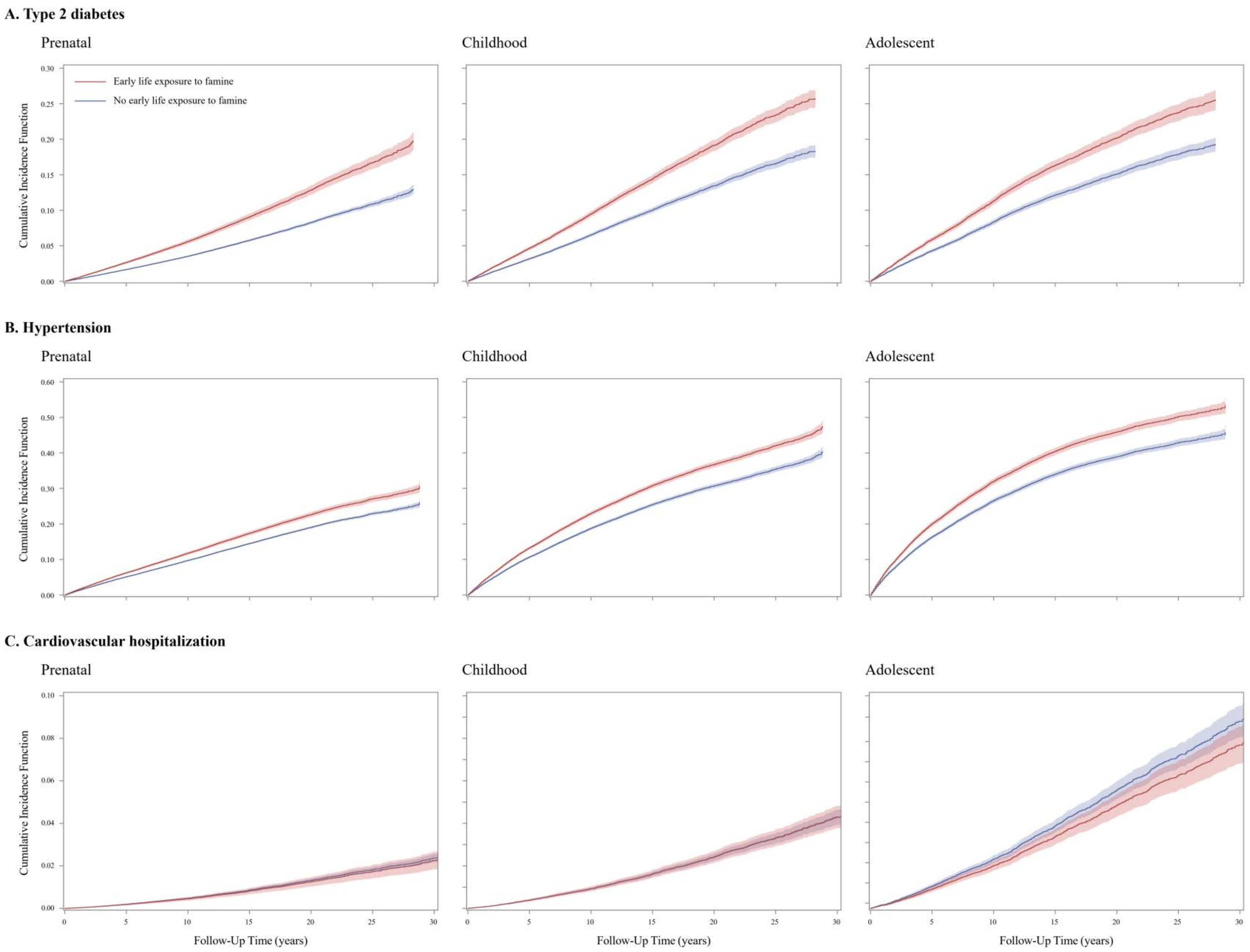

© 2025 Cao A et al. *JAMA Network Open.*

eFigure 5. Adjusted Cumulative Incidence Function Curves for Incident Type 2 Diabetes, Hypertension, and Cardiovascular Hospitalization

Three cumulative incidence function curves (prenatal, childhood, and adolescent exposure to famine) are shown on each row: type 2 diabetes (A; top row); hypertension (B; middle row) and cardiovascular hospitalization (C; bottom row)

© 2025 Cao A et al. *JAMA Network Open.*

eReferences.

1. Li, C. & Lumey, L. H. Early-Life Exposure to the Chinese Famine of 1959-1961 and Type 2 Diabetes in Adulthood: A Systematic Review and Meta-Analysis. *Nutrients* **14**, (2022).
2. Du, R. *et al.* Early-Life Famine Exposure and Risk of Cardiovascular Diseases in Later Life: Findings From the REACTION Study. *Journal of the American Heart Association* **9**, e014175 (2020).
3. Shu, Z. *et al.* Effects of fetal famine exposure on the cardiovascular disease risk in the metabolic syndrome individuals. *Diabetol Metab Syndr* **14**, 173 (2022).
4. Wang, Y., Jin, J., Peng, Y. & Chen, Y. Exposure to Chinese Famine in the Early Life, Adulthood Obesity Patterns, and the Incidence of Hypertension: A 22-Year Cohort Study. *Ann Nutr Metab* **77**, 109–115 (2021).
5. Xin, X., Yao, J., Yang, F. & Zhang, D. Famine exposure during early life and risk of hypertension in adulthood: A meta-analysis. *Critical Reviews in Food Science and Nutrition* **58**, 2306–2313 (2018).
6. Wang, C. *et al.* Association between fetal famine exposure and risk of type 2 diabetes: a prospective cohort study. *Applied Physiology, Nutrition, and Metabolism* vol. 47 321–327 (2022).
7. Yue, Q. *et al.* The Mediating Role of Systemic Inflammation in the Effects of Fetal Famine Exposure on Cardiovascular Disease in Adults: A Cohort Study. *The Journal of Nutrition* **153**, 1389–1397 (2023).
8. Cheng, M., Sommet, N., Kerac, M., Jopp, D. S. & Spini, D. Exposure to the 1959–1961 Chinese famine and risk of non-communicable diseases in later life: A life course perspective. *PLOS Global Public Health* **3**, e0002161 (2023).

© 2025 Cao A et al. *JAMA Network Open.*
