# Supplementary material for: Early Life Exposure to the Great Chinese Famine and Cardiometabolic Outcomes
Source: JAMA Netw Open. 2025 Nov 25;8(11):e2545444. doi: 10.1001/jamanetworkopen.2025.45444 (PMC12648349; doi:10.1001/jamanetworkopen.2025.45444)
Supplement: Supplement 2. — Data Sharing Statement [file jamanetwopen-e2545444-s002.pdf]

Data Sharing Statement

Cao. Early Life Exposure to the Great Chinese Famine and Cardiometabolic Outcomes. *JAMA Netw Open*. Published November 25, 2025. doi:10.1001/jamanetworkopen.2025.45444

Data

The dataset from this study is held securely in coded form at ICES. While legal data sharing agreements between ICES and data providers (e.g., healthcare organizations and government) prohibit ICES from making the dataset publicly available, access may be granted to those who meet pre-specified criteria for confidential access, available at http://www.ices.on.ca/DAS (email: das@ices.on.ca). The full dataset creation plan and underlying analytic code are available from the authors upon request, understanding that the computer programs may rely upon coding templates or macros that are unique to ICES and are therefore either inaccessible or may require modification.
